# Valorization of Ferulic Acid from Agro-Industrial by-Products for Application in Agriculture

**DOI:** 10.3390/polym14142874

**Published:** 2022-07-15

**Authors:** Maria Pilar Villanueva, Claudio Gioia, Laura Sisti, Laura Martí, Raquel Llorens-Chiralt, Steven Verstichel, Annamaria Celli

**Affiliations:** 1 AIMPLAS, Plastics Technological Centre, Gustave Eiffel, 4, 46980 Paterna, Valencia, Spain; pvillanueva@aimplas.es (M.P.V.); lmarti@aimplas.es (L.M.); rllorens@aimplas.es (R.L.-C.); 2 Department of Civil, Chemical, Environmental and Materials Engineering, University of Bologna, Via Terracini 28, 40131 Bologna, Bologna, Italy; laura.sisti@unibo.it (L.S.); annamaria.celli@unibo.it (A.C.); 3 Organic Waste Systems, Dok-Noord, 5, 9000 Gent, Belgium; steven.verstichel@ows.be

**Keywords:** ferulic acid, poly-dihydro (ethylene ferulate), antioxidant, poly (butylene succinate adipate), UV stabilizer, biodegradable mulch film

## Abstract

The use of bioplastic mulch in agriculture has increased dramatically in the last years throughout the world. Nowadays, biodegradable materials for mulching films strive to constitute a reliable and more sustainable alternative to classical materials such as polyethylene (PE). The main challenge is to improve their durability in the soil to meet the required service length for crop farming by using benign and sustainable antioxidant systems. Here, we report the design and fabrication of biodegradable materials based on polybutylene (succinate adipate) (PBSA) for mulching applications, incorporating a fully biobased polymeric antioxidant deriving from ferulic acid, which can be extracted from an industrial by-product. Poly-dihydro (ethylene ferulate) (PHEF) from ferulic acid was synthesized by a two-step polymerization process. It is characterized by improved thermal stability in comparison with ferulic acid monomer and therefore suitable for common industrial processing conditions. Different blends of PBSA and PHEF obtained by melt mixing or by reactive extrusion were prepared and analyzed to understand the effect of the presence of PHEF. The results demonstrate that PHEF, when processed by reactive extrusion, presents a remarkable antioxidant effect, even in comparison with commercial additives, preserving a high level of the mechanical properties of the PBSA matrix without affecting the biodegradable character of the blend.

## 1. Introduction

Plasticulture takes care of plastic materials’ use in agriculture and is a well-established practice since the 60s with objective benefits in crop yield and quality. Plastic mulch films have contributed to improve crop yields and productivity by increasing soil temperature, reducing water loss, improving soil fertility, and limiting weed growth [1,2,3]. Polyethylene (PE) is the most used polymer for such application, however, its complete removal after crop harvesting, is difficult, time-consuming, and expensive [4]; for this reason, every year a substantial amount of PE-based mulch remains in the fields, is eventually fragmented through photodegradation, and ends up being incorporated into the soil system through tillage. Even if collected, the film waste is highly contaminated with soil, sand, and organic material by up to 60–80% in weight of the total amount sold, making mechanical recycling economically unviable [4]. On the other hand, the removal of the mulching film causes the loss of an important superficial part of the soil against the principles of soil preservation, also indicated as a priority in EU policy [5].

In response to this issue, biodegradable mulch films have gained attention during the last decade, in agreement with the suggestions of the EU Commission that aims at developing a policy framework on bio-based, biodegradable, and compostable plastics and measures on the unintentional release of microplastics by 2022. With the ability to self-degrade at the end of the cycle, biodegradable films avoid the removal of plastics. The use of commercially available biodegradable-in-soil plastic materials (according to ISO 17556 and ISO 17033), such as Ecovio^®^M2351, MaterBi^®^EF04P, PHBHTMX131A, or BioPBSTM FD92PM, has been demonstrated to be an alternative to conventional materials [6,7]. These grades are based on biopolymers such as poly(butylene adipate-co-terephthalate) (PBAT), poly(lactic acid) (PLA), starch, polyhydroxyalkanoate (PHA), and poly(butylene succinate adipate) (PBSA). The tendency of these biodegradable materials to be consumed in environmental conditions raised the problem to modulate their degradation rate and adapting them to the specific agricultural application. In general, a key challenge for this class of materials is to improve their durability in the soil to meet the required service length for crop farming. As reported in the literature, biodegradable films break with elongations lower than 100%, when exposed to radiations higher than 150 kJ/m^2^ (i.e., around 2 months in the field) [8]. For example, PBAT embrittlement was observed in less than 4 weeks (i.e., 40 kJ/cm^2^) with an elongation at break of 30% [9]. Indeed, antioxidant and preserving systems for biodegradable mulching film applications must abide to severe specific requirements such as low ecotoxicity and inherent biodegradability. Recently, many efforts are devoted to the research of safe and natural antioxidants [10,11]. Carotenoids [12], vitamins [13,14,15], phenolic acids [16,17], flavonoids [18], stilbene structures [19], and even lignin [20,21] and tannins [22,23] represent the most notable classes of natural stabilizers.

Among the phenolic acids, ferulic acid (FA) has gained considerable attention in recent years for its use in preventing oxidative stress [24]. As a photoprotective and antioxidant agent, ferulic acid also prevents harmful radiation effects both as a UV absorber and a free radical scavenger [16,25,26]. Remarkably, H2020 Agrimax Project aims at extracting ferulic acid from agro-industrial by-products framing its production within cereal biorefinery [27]. Although to the best of our knowledge, no study was specifically directed towards the creation of materials for agriculture applications using FA, some remarkable papers report the incorporation of ferulic acid derivates into commonly used biodegradable polymers such as PLA, PBS, or starch [28,29,30]. In general, the main drawback of the use of FA is its limited thermal stability, which may cause extensive degradation during polymer melt processing. To overcome such limiting issues, FA can be protected by being incorporated into an organic-inorganic nanohybrid material [31,32,33,34,35,36], or chemically modified to synthesize stable additives [25,37].

A relatively new approach concerns the creation of ferulic acid-based macromolecular structures improving its thermal stability without sacrificing the antioxidant character [19]. The advantages of this still poorly explored method rely on the possibility to make blends between the matrix and FA-based antioxidant biopolymers thus avoiding premature leakages and undesired migration of the active compound. For example, crosslinked polymers with antioxidant properties were synthesized by inserting FA in a polymer based on methacrylic acid (MAA) and using ethylene glycol dimethyl acrylate as a comonomer and crosslinker. Copolymers of ferulic acid and methacrylic acid were also reported by Parisi et al. to present antioxidant and antifungal properties [38]. 

Framed in this scenario, this work aims to study the antioxidant and UV preserver effect of a ferulic acid-based polyester, namely poly-dihydro (ethylene ferulate), as for the second component in blends with biopolyesters, such as poly(butylene succinate adipate), specifically targeting mulching film applications in sustainable agriculture. 

Inspired by the principles of circular economy, this work strives to draw an industrially relevant process that valorizes ferulic acid as a high-value target from agroindustrial byproducts to obtain new bio-oligomers presenting antioxidant properties for biodegradable mulching films. 

## 2. Materials and Methods

### 2.1. Materials

Trans-Ferulic acid (≥99%) (FA), dibutyltin oxide (DBTO), and K_2_CO_3_ were supplied by Sigma Aldrich (Milan, Italy), H_2_ was supplied by Siad, palladium on carbon (10% on carbon, 55% water), and ethylene carbonate were supplied by TCI (Frankfurt, Germany). Poly(butylene succinate adipate), PBSA (BioPBSTM FD92PM), was supplied by PTT MCC Biochem Company (Banchang Town, Thailand) and was previously dehumidified at 50 °C for 5 h before its use. Luperox^®^101XL45 was used in this work as a crosslinking peroxide and was supplied by Arkema (Dusseldorf, Germany). This reagent was used for the crosslinking reaction between the PBSA and the oligomer PHEF produced in this work. Irganox^®^ 1010, as a commercial antioxidant additive, was acquired from BASF (Pontecchio Marconi, Italy). 

### 2.2. Synthesis of Methyl Dihydro Ferulate

Ferulic acid (600 g), Pd/C catalyst (40 g, 55% H_2_O), MeOH (3 L), and HCl (37%, 200 mL) were introduced, under inert atmosphere, in a 6 L three-neck round-bottom flask equipped with mechanical stirring, hydrogen inlet, and condenser. Initially, hydrogen was bubbled in the mixture, replacing the inert atmosphere, and the resulting mixture was heated at 60 °C for 24 h. The progression of the reaction was monitored by FTIR analysis by the disappearance of the band at 1633 cm^−1^ associated with the double bond and confirmed by ^1^H-NMR. The reaction was stopped after detecting the disappearance of the signal of the carboxylic acid by FT-IR analysis. After completion of the reaction, the methanol was removed under reduced pressure and the catalyst was filtered on a Celite pad to be recycled. Deionized water was added to the mixture and the product was obtained as a yellow dense liquid. The product was then separated and washed 3 times with water. All the aqueous phases reunited were extracted with 2 portions of 300 mL of ethyl acetate (EtOAc), collected, and dried with MgSO_4_. The resulting liquid was concentrated at reduced pressure. The product was obtained as a yellow liquid with a 95% yield and did not require additional purification.

### 2.3. Synthesis of Poly-Dihydro (Ethylene Ferulate)-PHEF

In a 250 mL reactor equipped with mechanical stirring and nitrogen inlet were subsequently introduced, methyl dihydro ferulate (140 g, 1 eq.), ethylene carbonate (64.5 g, 1.1 eq.), K_2_CO_3_ (700 mg), and dibutyl tin oxide (700 mg). The mixture was heated with an external bath at 180 °C for 2 h and maintained at 200 °C for 1.5 h. Then, the temperature was slowly raised to 230 °C while the pressure was gradually decreased to 0.1 mbar. The reaction mixture was kept in these conditions for 1 additional hour before being discharged. The resulting material was cooled until room temperature and ground for further uses. 

### 2.4. Preparation of PBSA/PHEF Compounds

PBSA blended with PHEF were produced in a Brabender Plasti Corder equipment, processing 55 g of material in each trial at 140 °C, at a roller speed of 60 rpm, and a mixing time of 2.5 min. Sheets of the material were prepared by compression molding at 170 °C and controlled cooling in a pressing machine. 0.8 mm thickness sheets were used to shape the tensile bars and perform the aging test. One physical blend of PBSA and PHEF was prepared with 5 wt% of the ferulic acid-based oligomer (PBSA_BLEND_) while two additional blends were reactively mixed in presence of 0.25 and 0.5 wt% of a radical source such as Luperox^®^ 101XL45 obtaining PBSA_REX0.25_ and PBSA_REX0.5_ respectively. A reference formulation was also prepared based on neat PBSA with 0.5 wt% of commercial Irganox^®^1010 as a common industrial stabilizer for long-term protection of polyolefins (PBSA_0.5IRG_). A maximum of 0.5 wt% of Irganox^®^ was used in the reference formulation following the recommendations of the technical datasheets of the product. 

### 2.5. NMR Analysis

^1^H-NMR spectra were recorded at room temperature on samples dissolved in CDCl_3_ using a Varian (Palo Alto, CA, USA) Mercury 400 spectrometer operating at 400 MHz for the proton and 100 MHz for carbon. Chemical shifts (δ) are reported in part per million with reference to chloroform solvent (CHCl_3_).

### 2.6. Gel Permeation Chromatography (GPC)

GPG measurements were performed by a HP 1100 Series equipped with a PL gel 5 µm Minimixed-C column. The instrument, provided with a Refractive Index and UV detectors, worked using chloroform as eluent. Polymer samples were dissolved in chloroform (at a concentration of 0.10% *w*/*v*) and a calibration plot was constructed with polystyrene standards.

### 2.7. Dynamic Scan Calorimetry (DSC)

The DSC analysis of the samples was studied employing a Perkin-Elmer (Waltham, MA, USA) DSC7 equipped with a liquid sub-ambient accessory, for experiments at low temperature. The instrument was calibrated using high purity standards and all the measurements were performed under nitrogen flow using sample masses of approximately 5 mg. The samples were heated from 25 °C to 200 °C at 20 °C/min and held at high temperature for 2 min to erase their previous thermal history. Then, they were cooled to −150 °C at 10 °C/min and finally, heated to 200 °C at 10 °C/min. The glass transition temperature (*T*_g_) was taken at the midpoint of the heat capacity increment during the second scan.

### 2.8. Thermogravimetric Analysis (TGA)

The analysis of the PHEF oligomer was performed with a Perkin–Elmer(Waltham, MA, US) TGA4000 thermo-balance under a nitrogen atmosphere (gas flow 40 mL/min) at 10 °C/min heating rate from 40 to 600 °C. The TGA of the PBSA formulations was performed by a thermogravimetric analyzer TGA Q5000 (TA Instruments). Heating from 20 to 600 °C at a heating ramp of 5 °C/min was applied in all the cases by using an air atmosphere. These tests were performed to measure the onset degradation temperature (*T*_onset_) and the oxidation induction time (OIT), following the procedure reported in the literature by Reano et al. [16].

### 2.9. Tensile Tests

Tensile tests were performed in a universal testing machine (Zwick model 1465 of 50 kN). Tensile properties were measured according to UNE-EN ISO 527-2 standard at a speed of 50 mm/min and by using small dog-bone bars (overall length 50 mm, width of ends 8.5 mm, length of the narrow portion 16 mm, width of narrow portion 4 mm). Parameters such as Young Modulus, stress, and elongation at break were obtained from the stress-deformation curves of five replicates registered during the tests. 

### 2.10. Aging Test

The resistance to UV radiation was evaluated with an aging test in a Xenotest chamber with an arc lamp following UNE EN ISO 4892-2 standard (test method A), in a test with a duration of 600 h and using dog-bone tensile bars exposed to a total ration of 129.6 MJ/m^2^ (between 300–400 nm). This radiation, according to the annual radiation in different worldwide areas such as Europe, Arizona, or Florida, has their equivalence to 7 months of outdoor exposure in Europe and around 4 months for Arizona and Florida. To evaluate the effect of the UV exposition, mechanical properties were tested after 0, 300, and 600 h of exposure. 

### 2.11. Biodegradability

The biodegradation in soil was evaluated according to ISO 17556 (2019) using standard soil. The standard soil consisted of a mixture of 70% industrial quartz sand, 10% kaolinite clay, 16% natural soil, and 4% mature compost. The soil was collected from a sandy field in Lokeren and 2 types of forest in Moerbeke (all located in Belgium). The mixture consisted of 1/3 field soil and 2/3 forest soil. The soil was sieved over a 2 mm sieve to remove stones and other inert materials, roots, and other plant debris, and thoroughly mixed. The mature compost was derived from the organic fraction of municipal solid waste. The waste was stabilized and aerated in a composting bin at the laboratory under controlled conditions for at least 20 weeks. Before use, the compost was sieved through a 5 mm sieve. Finally, salts were added to the standard soil employing nutrients solution (per l: KH_2_PO_4_ 9.6 g, MgSO_4_ 4.8 g, NaNO_3_ 19.2 g, urea 9.6 g, and NH_4_Cl 19.2 g/L) to obtain the final inoculum. At start-up, 2.0 g of reference material cellulose or test item PBSA_REX0.5_ was mixed with 500 g soil inoculum, while the control reactors contained only 500 g soil inoculum. The reactors were closed airtight and placed in the dark at 25 ± 2 °C. The evolved CO_2_ is absorbed in a beaker containing a KOH solution and determined by titration with a Metrohm 888 Titrando. The total test duration was 361 days.

## 3. Results and Discussions

### 3.1. Synthesis and Characterization of Poly-Dihydro (Ethylene Ferulate)-PHEF

**Figure 1:**
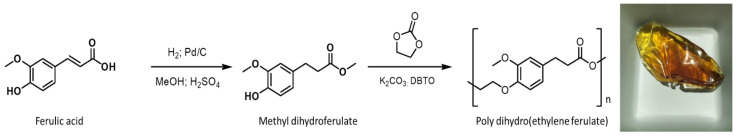
Synthesis of Poly-dihydro (ethylene ferulate) (PHEF).

Poly-dihydro(ethylene ferulate) (PHEF) was synthesized according to the two-step procedure described in Figure 1. First of all, ferulic acid (FA) was hydrogenated and esterified, to avoid undesired reactions during the polymerization step. Then, a one-pot etherification and polymerization of methyl dihydro ferulate with ethylene carbonate (Figure 1) was optimized according to a previously developed procedure for the synthesis of poly(ethylene vanillate) [39,40]. The ester functionality allows the ethylene carbonate to react selectively with the phenol and participates in the polymerization by transesterification only once the aliphatic hydroxyl is formed. The progress of the steps was followed by FT-IR analysis (Figure S1 in supplementary materials). The one-pot hydrogenation/esterification sees the decreasing of the double bond signals at 1620–1590 cm^−1^, and the shift of the signal related to the carbonyl moiety from 1660 to 1720 cm^−1^. The reaction with ethylene carbonate and the subsequent polymerization is associated to the complete consumption of phenolic groups at 3430 cm^−1^ along with a substantial modification of the footprint zone (1300–600 cm^−1^).

To the best of our knowledge, a previous two-step synthesis of PHEF was reported by Meier and co-workers [41], however, only oligomers presenting a *M*_w_ of 8000 Da were obtained after two purification steps. According to the procedure hereby proposed, a polyester presenting a molecular weight of 36,000 Da was obtained (Table 1; Figure S2 in supplementary materials). This approach allows avoiding toxic reagents and solvents usually exploited for functionalizing the phenolic moiety (i.e., halogenated compounds), to meet the requirements of green chemistry [41].

The ^1^H-NMR analysis of PHEF reported in Figure S3 (in supplementary materials) shows two signals at 4.4 and 4.2 ppm related to the new ethylene structure in the polymeric chain, introduced by the ethylene carbonate while the disappearance of the methyl ester signal at 3.6 ppm is a clear indication of a complete polymerization. The ^1^H-NMR analysis did not evince any additional signal associable with undesired side reactions occurring during the polymerization.

The synthesized PHEF has been characterized in terms of thermal behavior. The TGA analysis (Figure S4 in supplementary materials) conducted in the air atmosphere demonstrates that PHEF starts its thermo-oxidative degradation at 380 °C (T_Onset_) with a maximum degradation rate (T_max_) at 423 °C (Table 1). In the same conditions, ferulic acid starts to degrade at 206 °C with a T_max_ of 245 °C. This outstanding difference demonstrates that the presence of covalent bonds between the monomeric units and the absence of the double bonds give PHEF enhanced stability; the polymer can be processed with common industrial techniques in presence of other biopolymers. DSC analysis (Figure S5 in supplementary materials) shows that PHEF is an amorphous material presenting a *T*_g_ of about 30 °C, suitable to act as a plasticizer in blend with rigid biopolymers, to reduce their glass transition temperature. Most likely, the flexible aliphatic segment deriving from the ethylene carbonate residue along with the hydrogenated double bond contributes to destabilizing the aromatic interactions producing a detrimental effect on the crystallinity of the system.

### 3.2. Effect of PHEF on Thermal and Antioxidant Properties of PBSA

PHEF was tested as a second component of a polymeric blend, obtained by physical mixing or reactive extrusion, with PBSA, as reported by the composition in Table 2. The presence of a certain amount of PHEF as a second component can be useful to modify the properties of the main constituent of the blend. Now, the focus is to investigate antioxidant properties, but other property variations can be found.

TGA tests were performed, under oxygen atmosphere, to measure the oxidation induction time (OIT) as an indication of the preservative effect of PHEF on PBSA structure. Pristine PBSA and PBSA additivated with 0.5 wt% of Irganox^®^ 1010 were tested as reference materials (Table 2). The TGA curves reported in Figures S6 and S7 (in supplementary materials), and Table 2, show that, in the presence of just 5% of PHEF, the blends increased their respective OIT of about 13–15 min in comparison with neat PBSA, while their T_onset_ increased up to 67–76 °C. Remarkably, these enhancements resulted even more significant than the ones observed with commercial antioxidant Irganox^®^1010 which increased the OIT of about 8.5 °C with a T_onset_ of 325 °C. 

Apparently, the samples prepared by reactive extrusion, with the crosslinking agent, do not have a significant variation in the thermal stability in comparison to the sample without crosslinking agent (physical blend). Therefore, PHEF seems very efficient in delaying the oxidative degradation process of PBSA independently of the methodology used in the preparation of the blends (physical or reactive extrusion).

### 3.3. Aging Tests: Effect of PHEF as UV Radiation Stabilizer

**Figure 2:**
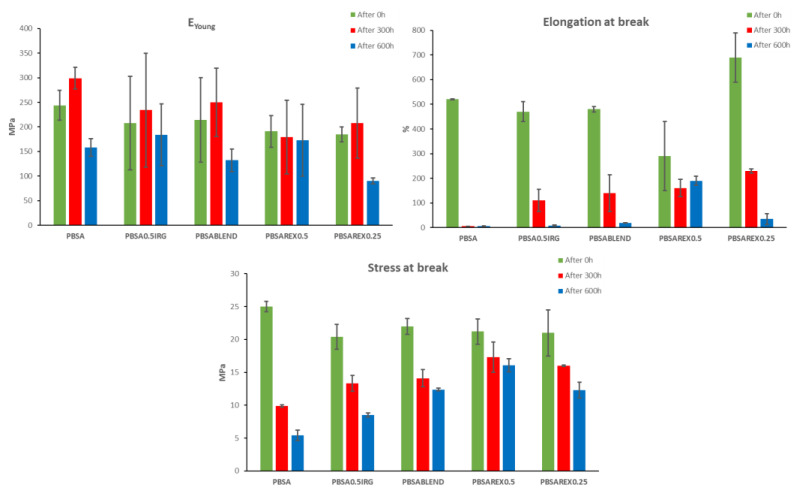
Graphical summary of the evolution of the mechanical performances of the samples during the aging test.

The effectiveness of PHEF as a UV stabilizer component was further tested by performing an accelerated aging test of the obtained samples and studying the evolution of their mechanical performances over time by tensile tests. Dog-bone bars of the different material formulations were submitted to a total radiation of 129.6 MJ/m^2^ corresponding to a time of exposure of 6 months (equivalent to 7 months in Europe). Samples were analyzed after 0 h, 300 h, and 600 h of exposure and mechanical testing were carried out. Young modulus, stress, and elongation at break were measured before the aging test started and after each extraction (Figure 2, Table 3).

As reported in Table 3, the mechanical performances of the samples before irradiation (t = 0 h) showed that the unique addition of Irganox^®^ or PHEF in a physical blend of PBSA did not have a significant modification on the mechanical properties of neat PBSA, since the Young Modulus (E_Young_), the stress (σ_break_), and strain (ε_break_) at break are only slightly lower respect to the reference sample. In the case of PHEF, this trend could be ascribable to a plasticizing effect of this component. On the contrary, the samples prepared by reactive extrusion present a different behavior depending on the amount of Luperox^®^ employed in fact, a low concentration of the radical source enhances the flexibility of the material while a higher concentration drastically reduces its ductility, probably caused by a partial reticulation of the material. 

After 300 h of accelerated UV exposition (see Figure 2), the pristine PBSA showed a stress at break of 9.9 MPa and an elongation at break of 5.4%, testifying the severe degradation of all the material performances. As expected, the presence of the commercial antioxidant additive (Irganox^®^ 1010) partially undermined the degradative processes and allowed the material to partially retain its flexible behavior. At the same time, all the samples blended with PHEF demonstrated a similar trend but, the one obtained by reactive blending with a low Luperox^®^ content kept a quite high elongation at break.

The results obtained after 600 h of exposition further confirmed a drop in performances for PBSA and PBSA_0.5IRG_ reaching a stress at break of 5.4 and 8.5 MPa and an elongation at break of 6.4 and 8.1% respectively. In the same conditions, PHEF-based blends presented better mechanical performances. In particular, PBSA_REX0.5_ retained a stress at break of 16.1 MPa while presenting a 190% of elongation at break, indicating that for longer exposition a higher degree of reactions of the material is preferable.

### 3.4. Biodegradability Results

**Figure 3:**
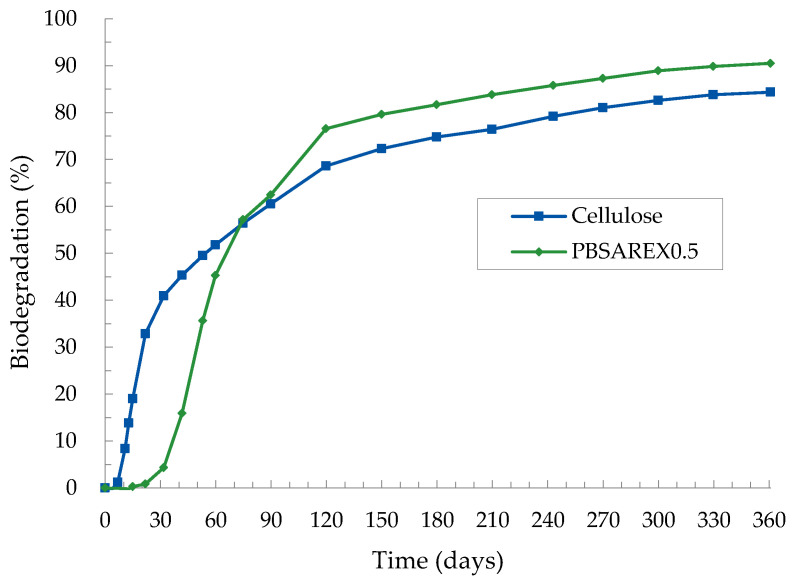
Biodegradation curve in soil conditions according to ISO 17556.

The most suitable sample selected (PBSA_REX0.5_) was finally submitted to biodegradability tests in the soil environment. The behavior of this sample was compared to a cellulose reference material as indicated in the international standard for biodegradation in soil. According to ISO 17556 (2019), the degree of biodegradation of the reference material (the cellulose sample) should be more than 60% at the plateau phase or the end of the test. In the test performed in this study, after 90 days, a biodegradation percentage of 60.5% was measured for reference material cellulose and after 361 days a plateau in biodegradation was reached at a level of 84.3% (Figure 3). The biodegradation of PBSA_REX0.5_ started after a lag phase of about 1 month, during which the microorganisms were adapted to the polymer, and proceeded at a high rate. After 90 days a biodegradation of 62.4% was measured. The biodegradation continued at a moderate rate to reach a plateau in biodegradation at the end of the test at a value of 90.5%. On a relative basis, compared to suitable reference material cellulose, a biodegradation of 107.3% was calculated. According to EN 17033 Plastics-Biodegradable mulch films for use in agriculture and horticulture-Requirements and test methods (2018) a material must reach ≥90% absolute or relative biodegradation in the soil at ambient temperature within 24 months. This criterion was fulfilled for our formulation PBSA_REX0.5_, containing 5% of PHEF and 0.5% of the crosslinking agent.

## 4. Conclusions

A biobased polymer such as PHEF was synthesized from ferulic acid through a two-step procedure. The resulting structure highlighted high thermal stability, demonstrating to be suitable for common industrial processing such as extrusion. PHEF/PBSA blends, fabricated by melt mixing and reactive extrusion, demonstrated enhanced oxidation induction time as well as the T_onset_, thus outperforming even commercially available additives such as Irganox^®^. The observed antioxidant effect is particularly remarkable considering that PHEF does not present free phenolic compounds, usually addressed for free radical stabilization, opening a new perspective for future mechanistic studies.

The evolution of the mechanical performances of the blends was studied under accelerated photodegradation conditions. While un-stabilized PBSA demonstrated a predictable fast decrement of both stress and elongation at break, the presence of PHEF managed to interfere with the oxidation processes preserving the matrix. 

Finally, biodegradation tests in soil confirmed that the biobased ferulic acid derivative is not interfering with the natural degradation processes and represents a potential additive for agriculture, fulfilling the biodegradation requirement of EN 17033 on biodegradable mulch films.

Framed in the perspective of sustainable agriculture, the results herby reported open new insights for the synthesis of fully biobased, sustainable, and biodegradable additives presenting the dual effect as antioxidants and UV preservers, improving, and modulating the shelf-life of mulching films.

## Figures and Tables

**Table 1:** Summary of the properties of PHEF.

T_Onset_ (°C) ^1^	T_max_ (°C) ^1^	T_g_ (°C) ^2^	M_w_ (Da) ^3^	PDI ^3^
380	423	30.7	36,000	2.3

^1^ Obtained by TGA in air atmosphere; ^2^ Obtained by DSC analysis; ^3^ Obtained by GPC analysis.

**Table 2:** Composition of the tested materials and their thermal performances under oxidative environment.

**Sample**	**PBSA (%)**	**PHEF (%)**	**Luperox^®^ 101XL45 (%)**	**Irganox^®^ 1010 (%)**	**OIT (min)**	**T_Onset_ (°C)**
PBSA	100	--	--	--	49.3	284
PBSA_0.5IRG_	99.5	--	--	0.5	57.8	325
PBSA_BLEND_	95	5	--	--	63.1	351
PBSA_REX0.5_	94.5	5	0.5	--	63.1	353
PBSA_REX0.25_	94.75	5	0.25	--	64.4	360

**Table 3:** Mechanical properties of PBSA formulations at initial stage (t = 0 h) and after 300 h and 600 h of aging test. All the values here reported were obtained from the average value of 5 tests along with their standard deviation.

Time of Irradiation	0 h			300 h			600 h		
Sample	E_Young_ (MPa)	σ_break_ (MPa)	ε_break_ (%)	E_Young_ (MPa)	σ_break_ (MPa)	ε_break_ (%)	E_Young_ (MPa)	σ_break_ (MPa)	ε_break_ (%)
PBSA	244 ± 30	25.0 ± 0.8	520 ± 2	299 ± 22	9.9 ± 0.2	5.4 ± 0.2	158 ± 18	5.4 ± 0.8	6.4 ± 0.3
PBSA_0.5IRG_	208 ± 95	20.4 ± 1.9	470 ± 40	234 ± 116	13.3 ± 1.2	110 ± 45	184 ± 63	8.5 ± 0.3	8.1 ± 1.5
PBSA_BLEND_	214 ± 86	22.0 ± 1.2	480 ± 11	250 ± 69	14.1 ± 1.3	140 ± 74	132 ± 23	12.4 ± 0.2	19.0 ± 1.0
PBSA_REX0.5_	191 ± 32	21.2 ± 1.9	290 ± 140	179 ± 75	17.3 ± 2.3	160 ± 35	173 ± 73	16.1 ± 1.0	190.0 ± 18.0
PBSA_REX0.25_	185 ± 15	21.0 ± 3.5	690 ± 100	208 ± 71	16.0 ± 0.1	230 ± 8	90.3 ± 6.1	12.3 ± 1.2	35.0 ± 22.0

Note: all the values included represent the average and the standard deviation.

## Data Availability

Not applicable.

## Supplementary Materials

The following supporting information can be downloaded at: https://www.mdpi.com/article/10.3390/polym14142874/s1, Figure S1: FT-IR of ferulic acid (FA), Methil dihydroferulate, and Poly-dihihidro (ethylene ferulate) (PHEF); Figure S2: SEC analysis of PHEF; Figure S3: ^1^H-NMR of Poly-dihihidro (ethylene ferulate) (PHEF); Figure S4: TGA of ferulic acid (FA), and Poly-dihihidro (ethylene ferulate) (PHEF) under air atmosphere; Figure S5: DSC analysis of Poly-dihihidro (ethylene ferulate) (PHEF); Figure S6: TGA curves of PBSA formulations: weight loss vs. time; Figure S7: TGA curves of PBSA formulations: weight loss vs. temperature.
