# Supplementary material for: Valorization of Ferulic Acid from Agro-Industrial by-Products for Application in Agriculture
Source: Polymers (Basel). 2022 Jul 15;14(14):2874. doi: 10.3390/polym14142874 (PMC9325307; doi:10.3390/polym14142874)
Supplement: Supplementary file 1 [file polymers-14-02874-s001.zip › polymers-1773761-supplementary.pdf]

Supplementary material

Valorization of Ferulic Acid from Agro-Industrial by-Products for Application in Agriculture

Maria Pilar Villanueva [1], Claudio Gioia [2,*], Laura Sisti [2], Laura Martí [1], Raquel Llorens-Chiralt [1], Steven Verstichel [3] and Annamaria Celli [2]

[1] AIMPLAS, Plastics Technological Centre, Gustave Eiffel, 4, 46980 Paterna Valencia, Spain; pvillanueva@aimplas.es (M.P.V.); lmarti@aimplas.es (L.M.); rllorens@aimplas.es (R.L.-C.)
[2] Department of Civil, Chemical, Environmental and Materials Engineering, University of Bologna,
Via Terracini 28, 40131 Bologna, Italy; laura.sisti@unibo.it (L.S.); annamaria.celli@unibo.it (A.C.)
[3] Organic Waste Systems, Dok-Noord, 5, 9000 Gent, Belgium; steven.verstichel@ows.be
* Correspondence: claudio.gioia2@unibo.it

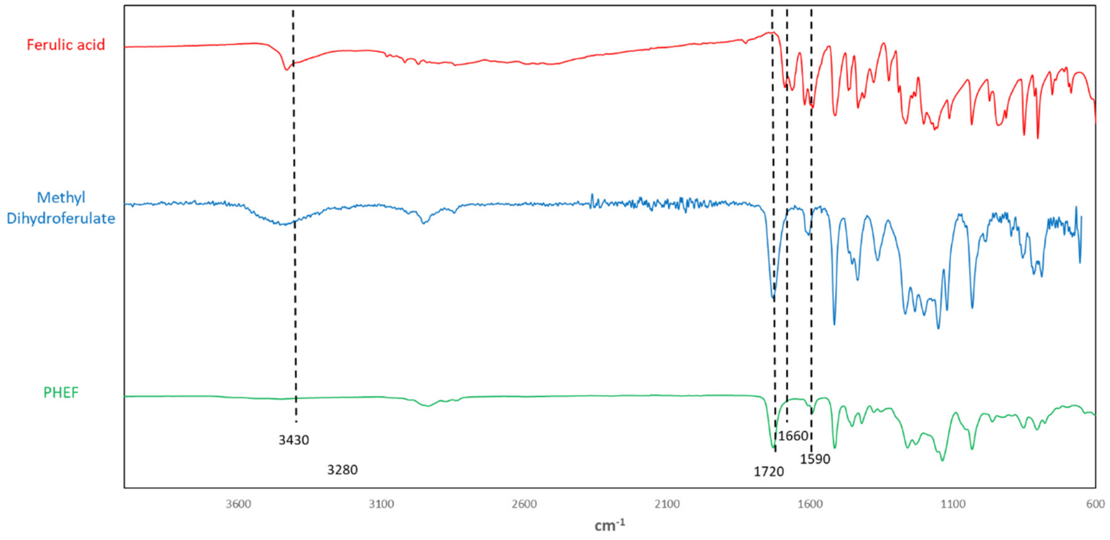

Figure S1. FT-IR of ferulic acid (FA), Methil dihydroferulate, and Poly-dihihidro (ethylene ferulate) (PHEF).

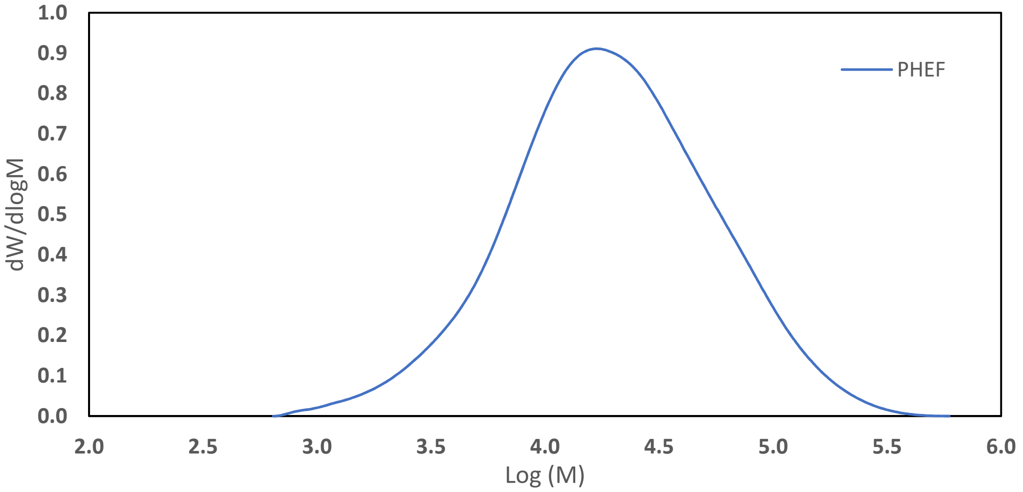

Figure S2. SEC analysis of PHEF

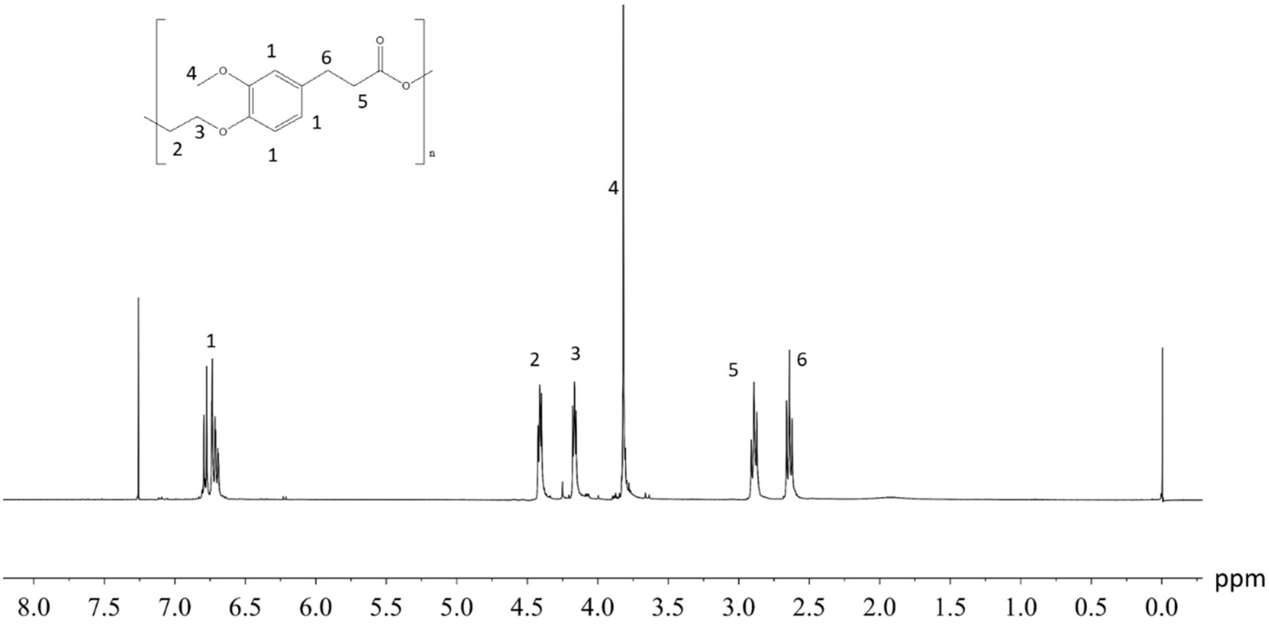

Figure S3. ^{1}H-NMR of Poly-dihihidro (ethylene ferulate) (PHEF).

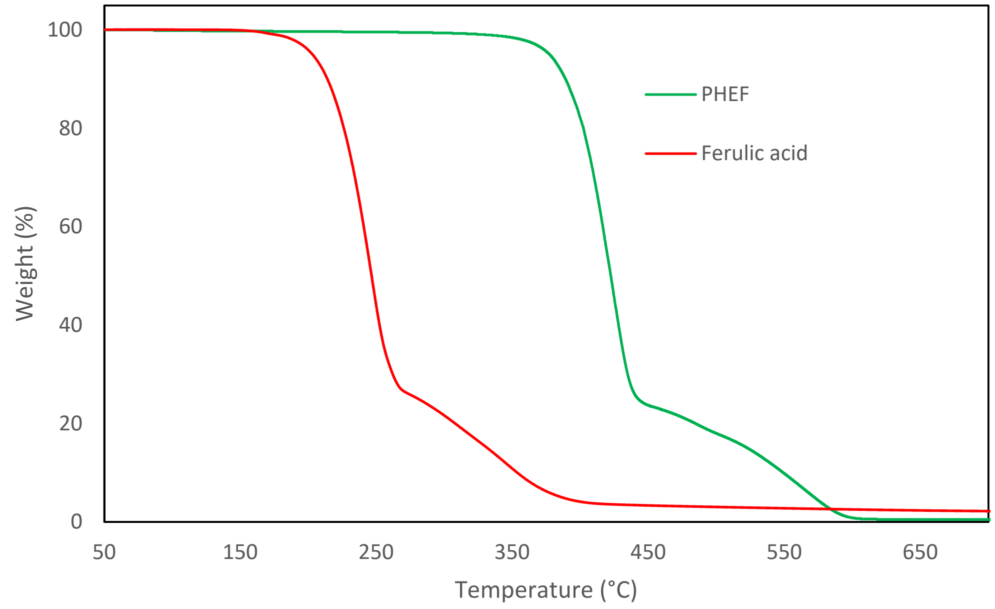

Figure S4. TGA of ferulic acid (FA), and Poly-dihihidro (ethylene ferulate) (PHEF) under air athmosphere.

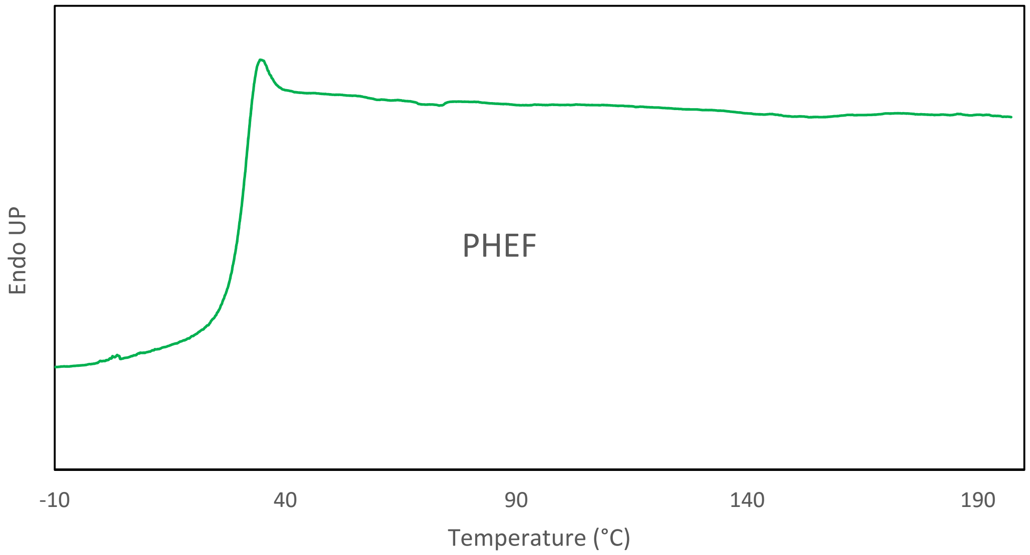

Figure S5. DSC analysis fo Poly-dihihidro (ethylene ferulate) (PHEF).

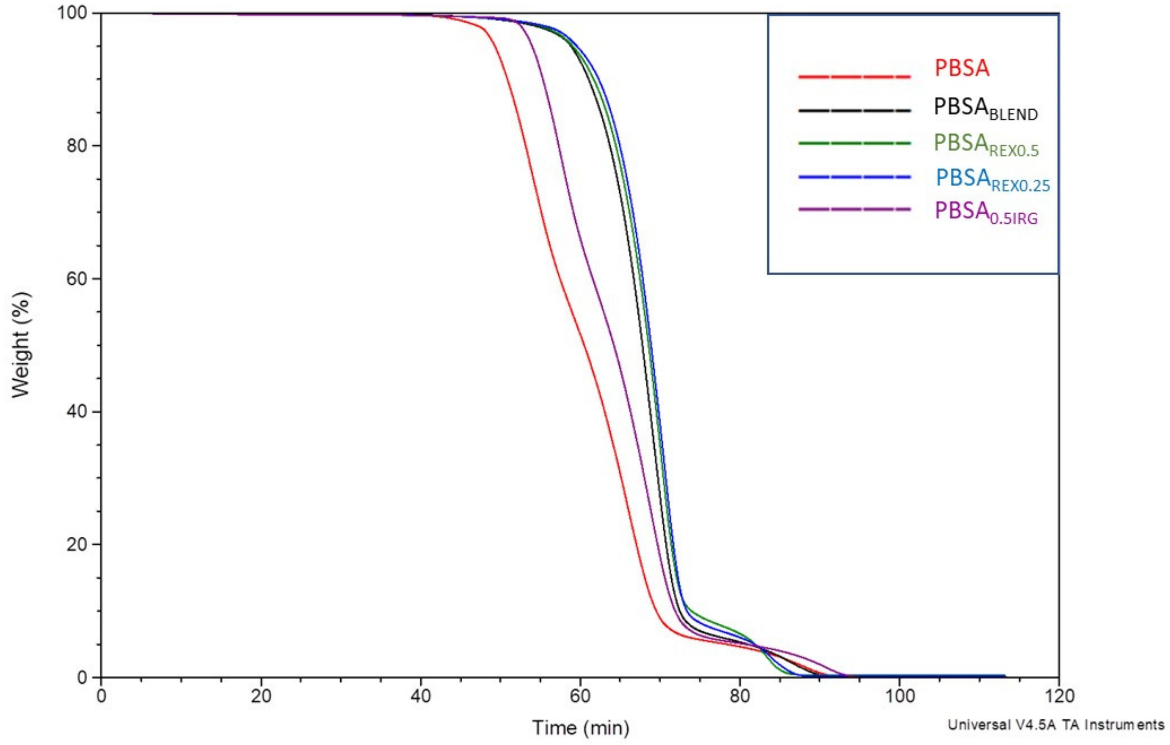

Figure S6. TGA curves of PBSA formulations: weight loss vs time.

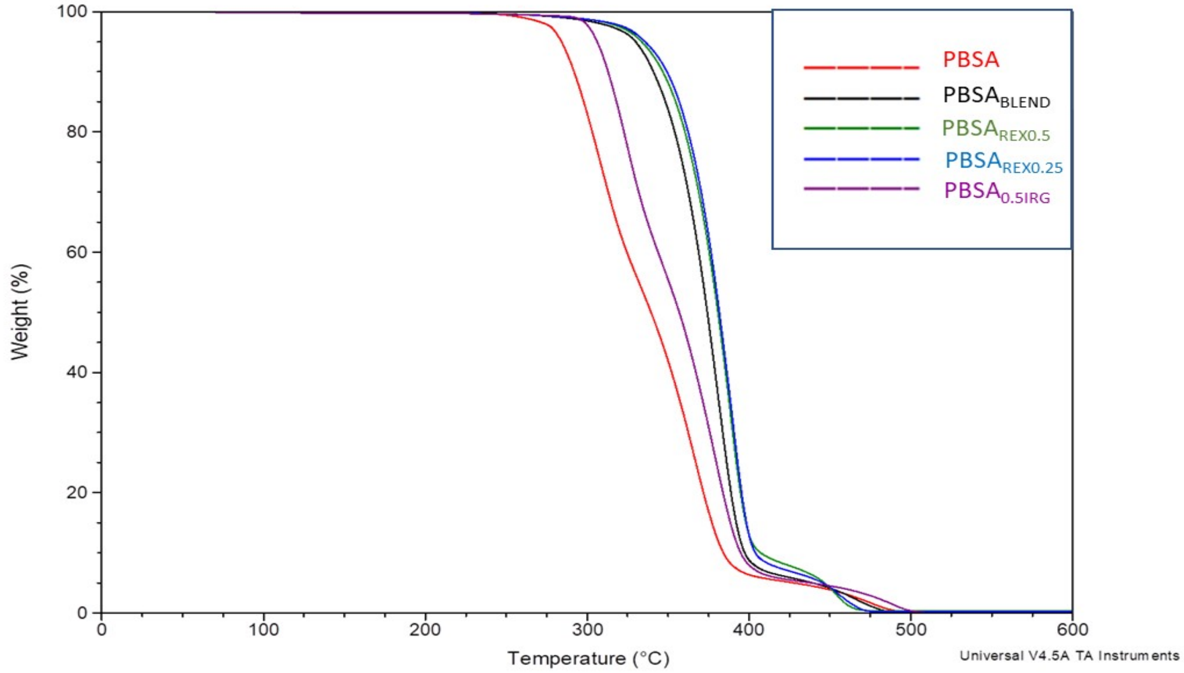

Figure S7. TGA curves of PBSA formulations: weight loss vs temperature.
